# Thymoquinone Produces Cardioprotective Effect in β-Receptor Stimulated Myocardial Infarcted Rats via Subsiding Oxidative Stress and Inflammation

**DOI:** 10.3390/nu14132742

**Published:** 2022-06-30

**Authors:** Sumit Rathod, Yogeeta Agrawal, Abdulla Sherikar, Kartik T. Nakhate, Chandragouda R. Patil, M. F. Nagoor Meeran, Shreesh Ojha, Sameer N. Goyal

**Affiliations:** 1 Shri Vile Parle Kelavani Mandal’s Institute of Pharmacy, Dhule 424001, Maharashtra, India; sumit.rathod@svkm.ac.in (S.R.); yogeeta.goyal@svkm.ac.in (Y.A.); abdulla.sherikar@svkm.ac.in (A.S.); kartik.nakhate@svkm.ac.in (K.T.N.); 2 Department of Pharmacology, R. C. Patel Institute of Pharmaceutical Education and Research, Shirpur 425405, Maharashtra, India; crpatil@rcpatelpharmacy.co.in; 3 Department of Pharmacology and Therapeutics, College of Medicine and Health Sciences, United Arab Emirates University, Al Ain P.O. Box 15551, United Arab Emirates; nagoormeeran1985@uaeu.ac.ae; 4 Zayed Bin Sultan Center for Health Sciences, United Arab Emirates University, Al Ain P.O. Box 15551, United Arab Emirates

**Keywords:** myocardium, glutathione, pro-inflammatory mediators, antioxidant, hemodynamic

## Abstract

Earlier studies reported that long-term treatment with thymoquinone (TQ) at a high dose (20 mg/kg) exerts a cardioprotective effect against isoproterenol (ISO)-triggered myocardial infarction (MI) in rats. In the present study, we tested the hypothesis that TQ, as a potent molecule, can exhibit cardioprotective effects at the lower dose for a short-term regimen. The rats were administered with TQ (5 mg/kg, intraperitoneal) at the 4 h interval for 2 days. ISO (100 mg/kg/day, subcutaneous) was given for 2 days to produce MI. ISO challenge results in deformation in ECG wave front, elevated left ventricular (LV) end-diastolic pressure, and reduced LVdP/dtmax and LVdP/dtmin. The levels of the cardiac biomarker in serum, such as creatine kinase MB, alanine aminotransferase, and aspartate aminotransferase, were increased. In the myocardium, a rise in malonaldehyde and decreased superoxide dismutase, glutathione, and catalase contents were observed. Furthermore, increased levels of tumor necrotic factor-α, interleukin-6, and interleukin-1β were observed in the myocardium. TQ pretreatment significantly normalized alterations in hemodynamic parameters, strengthened the antioxidant defense system, and decreased the contents of pro-inflammatory cytokines and hepatic enzymes as compared to the ISO group. Based on the results, TQ appears to be cardioprotective at low doses, and effective even administered for a shorter duration.

## 1. Introduction

Globally, coronary heart disease (CHD) is the leading cause of morbidity and mortality. Myocardial infarction (MI) is the most common form of CHD [1]. Further, the most important hallmark of MI is heart failure which is caused by poor blood supply to the heart or deteriorating hemodynamics, resulting in cardiac death or severe hemodynamic impairment [2]. Heart failure is linked to elevated levels of certain biochemical parameters such as high lipid profile, and blood sugar along with several biological changes including high blood pressure, obesity, and aging [3]. The non-selective adrenoreceptor agonist isoproterenol (ISO) is known to trigger oxidative stress in the cardiac tissues, resulting in high-level tissue necrosis. Additionally, it increased chronotropic, inotropic, and Ca^2+^ overloads. In the process, oxidative catecholamine metabolism produced an excess of free radicals, resulting in a loss of plasma membrane integrity and the inactivity of cardiac enzymes, such as aspartate aminotransferase (AST) and alanine aminotransferase (ALT) [3].

MI induction in rodents by ISO is the widely used paradigm for studying the protective function of several compounds [4]. Previous research demonstrated that ISO causes significant biochemical and morphological changes akin to MI in humans, resulting in cell destruction and necrosis. Additionally, ISO is connected to the onset of oxidative damage, lipid peroxidation, impaired hemodynamics, and left ventricular function following hypertrophy, inflammatory cell infiltration, and fibrosis [5]. Thymoquinone (TQ), also known as 2-isopropyl-5-methylbenzo-1, 4-quinone, is a biologically active phytochemical derived from the seeds of *Nigella sativa*, a member of the *Ranunculaceae* family. The seeds contain volatile oil of 18.4-24% and fixed oil of 30% along with some important monoterpenes, mainly p-cymene and ct-pinene [6]. A previous study showed that the volatile oil of *Nigella sativa* has been found to lower cardiac activity and blood pressure [7]. TQ has also offered beneficial properties, including anticancer, antioxidative, hepatoprotective, anti-hypertensive, and antidiabetic [8,9]. TQ exerts cardiac muscle relaxant, and vasodilator effects by inhibiting the influx of Ca^2+^ ions mediating voltage-gated Ca^2+^ channels. TQ was also shown to prevent myocardial reperfusion and ischemia-induced arrhythmia [10].

In our previous study, we reported the chronic effect of a relatively high dose of TQ administered orally for 21 days (20 mg/kg) to the rats subjected to the development of myocardial injury following the administration of ISO [11]. However, in another study, following a similar chronic protocol including dose and route of administration, TQ exerted cardioprotective effects [12]. TQ in a rat model of ISO-induced MI exerts a cardioprotective effect by modulating cytochrome c activity and MMP-9 expression [13]. In another study, the long-term administration of oral TQ induced a positive inotropic effect in rats [14]. However, in a very recent study, the acute effects of TQ showed to exert cardioprotective effects on ventricular myocytes, and suggested as a therapeutic agent in diabetic cardiomyopathy and cardiac hypertrophy, which involves the over-activated β-adrenergic system [15]. The potent activity of TQ encouraged us to investigate its acute effects in the ISO-induced experimental animal model of MI. In the present study, we chose a 5 mg/kg intraperitoneal (i.p.) dose of TQ, which is smaller than 1/10th of its LD_50_ (57.5 mg/kg) in rats. We explored the acute effects of a safe dose of TQ [16,17]. Considering the cardiovascular effects of chronic oral doses of TQ in previous studies, the current investigation studied the myocardial protective action of TQ at the lowest safe dose in acute MI induced by ISO in rats. TQ at a low dose was administered to rats at certain intervals to demonstrate its therapeutic effect in MI.

## 2. Materials and Methods

### 2.1. Subjects

Four-to-six-week-old adult male Wistar rats weighing 200–250 g were used. A 12:12 light-dark cycle, 24 ± 2 °C room temperature, and 35–60% humidity were employed in the animal house facility. The regular pellet food and water were provided on an ad libitum basis. The Institutional Animal Ethical Committee of RCPIPER, Shirpur, Maharashtra, India, approved the research proposal (approval no. IAEC/CPCSEA/RCPIPER/2017).

### 2.2. Chemicals

Sigma-Aldrich supplied the ISO and TQ (St. Louis, MO, USA). Furthermore, ERBA diagnostics supplied creatine kinase MB (CK-MB), serum glutamic oxaloacetic transaminase (SGOT), and serum glutamic pyruvic transaminase (SGPT) assays (Mannheim, Germany). ELISA kits were purchased from e-Bioscience (San Diego, CA, USA) to measure tumor necrosis factor-alpha (TNF-α), interleukin-6 (IL-6), and interleukin-1-beta (IL-1β) levels in cardiac tissue homogenate. For the administration to rats, ISO and TQ were dissolved in normal saline and olive oil (Figaro, Spain) respectively. GSH, CAT, SOD, and MDA were determined by using standard kits from e-Bioscience (San Diego, CA, USA). Most other reagents utilized in this investigation were of analytical grade.

### 2.3. Experimental Groups

**Figure 1:**
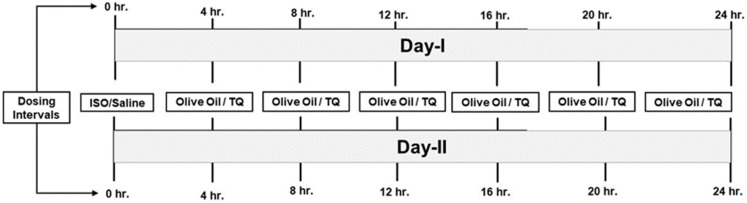
Treatment schedule. hr., hours; ISO, isoproterenol; TQ, thymaquinone.

Twenty-four rats were used in the present study. They were divided into four groups (n = 6 per group) and subjected to various treatments (Figure 1).

- Group 1 (control) (olive oil + saline): olive oil (0.2 mL, i.p.) was administered at an interval of 4 h for 48 h (2 days), i.e., 6 multiple doses per day. In parallel, on days 1 and 2, normal saline (s.c.) was administered at an interval of 24 h.
- Group 2 (olive oil + isoproterenol): olive oil (0.2 mL, i.p.) was administered at an interval of 4 h for 2 days. In parallel, on days 1 and 2, ISO (100 mg/kg, s.c.) was administered at an interval of 24 h.
- Group 3 (thymoquinone + saline): TQ (5 mg/kg, i.p.) was administered at an interval of 4 h for 48 h, i.e., 6 multiple doses per day. This dosing strategy was adopted since the plasma half-life of TQ is 3.5–4 h [18]. Since we were interested in studying the effects of the lower and safer dose of TQ, a multiple-dosing regimen was essential. In parallel, on days 1 and 2, normal saline (s.c.) was administered at an interval of 24 h.
- Group 4 (thymoquinone + isoproterenol): TQ (5 mg/kg, i.p.) was injected at an interval of 4 h for 48 h. Two doses of ISO (100 mg/kg) were administered on days 1 and 2 at an interval of 24 h.

### 2.4. Hemodynamic Parameters

#### 2.4.1. ECG Monitoring

Rats were anesthetized with urethane and put on an animal surgical board in the supine position. Based on previous studies, three-needle electrodes were inserted into each forelimb and hind limb in this procedure, with one positive and one negative electrode aimed towards the heart, whereas neutral electrodes were positioned on the hind limb [19]. The electrodes were linked to the Power Lab’s data-collecting equipment supplied by AD Instruments, Sydney (Sydney, Australia).

#### 2.4.2. Blood Pressure and Left Ventricular Pressure Monitoring

The surgical process, along with the recording of hemodynamic parameters, was based on the previously demonstrated technique. Precisely, in this procedure, rats were anesthetized with the i.p. administration of urethane (1.25 g/kg) 24 h after ISO exposure. Euthanized rats were kept on the surgery board to perform tracheotomy by opening neck-through ventral middle incision. A poly-ethane tube was used with the required diameter specification to perform right carotid artery cannulation using a three-way cannula (0.30 mm × 0.40 mm internal; outer diameter). Furthermore, a heparinized saline solution was filled into a cannula and connected to the data acquisition system of Power Labs. The heart rate (HR), systolic arterial pressure (SAP), diastolic arterial pressure (DAP), and mean arterial pressure (MAP) were measured using a pressure transducer [20].

To assess hemodynamic parameters of the left ventricular, a wide bore sterile metal cannula (1.5 mm diameter) coupled to a pressure transducer filled with heparinized saline (50 U/mL) was employed. On a Power Lab data acquisition system, the left ventricular pressure (LVEDP) and the maximum rate of rising and falling of the left ventricular pressures (peak + LVdP/dtmax and peak − LVdP/dtmax)] were recorded [21].

To record hemodynamic data, rats from each group were euthanized with i.v. sodi-um pentobarbitone (100 mg/kg). The heart tissues were removed and examined biochemically and histologically. Hearts were frozen in liquid nitrogen to perform biochemical analysis, and then fixed in 10% neutral buffered formalin for light microscopic investigations.

### 2.5. Biochemical Parameters

#### 2.5.1. Serum and Tissue Homogenate Preparation

Similarly, the blood was collected from the heart via cardiac puncture; the collected blood was transferred immediately into ice-cold 10 mL sterile test tubes. The serum was separated by centrifuging the collected blood for 5 min at 1200× *g* and room temperature (RT). The apex regions of the heart were removed from liquid nitrogen, and homogenized in 50 mM, pH 7.4, and ice-cold phosphate buffer solution. Centrifugation was performed for 20 min, at 7000 RPM (2739× *g*) at 4 °C, and supernatant produced was used to determine tissue parameters.

#### 2.5.2. Estimation of Protein Concentration

The protein concentration was determined using previously reported studies [22]. In the methodology, 10 mL of tissue’s supernatant was made up to 100 mL with 1N NaOH, vortexed and the absorbance was measured at 595 nm using the Bradford reagent. The protein content was evaluated using a standard curve constructed from known values of commercial products from Bovine Serum Albumin (Sigma Chemicals, St. Louis, MO, USA).

#### 2.5.3. Estimation of Oxidative Biomarkers

As described in the earlier studies, the oxidative parameters such as GSH [23], CAT [24], SOD [25], and MDA [26] were estimated using standard kits from e-Bioscience (San Diego, CA, USA).

#### 2.5.4. Estimation of CK-MB

The CK-MB isoenzyme assessment in serum was performed using a commercially available kit (ERBA diagnostic, Mannheim, Germany) and based on previously reported studies [27]. In the present investigation, the mean absorbance change per time (ΔA/min) was estimated. At pH 7.4 and 30 °C, one unit of CK-MB was demarcated as the quantity of enzyme necessary to convert 1 mol of phosphate from phosphocreatine to ADP per min. The content of enzymes was represented as IU/mg protein.

#### 2.5.5. Estimation of TNF-α, IL-6 and IL-1β

The homogenate of heart tissue was employed for the assessment of proinflammatory cytokines. The assessment of rat TNF-α, IL-6, and IL-1β was performed using a commercially available ELISA kit (B.D. Biosciences Pharmingen, Bedford, MA, USA) [28,29]. At 450 nm, samples were spectrophotometrically measured.

#### 2.5.6. Estimation of SGPT (ALT) and SGOT (AST)

SGPT and SGOT levels in serum were determined using commercial kits (ERBA diagnostic Mannheim, Germany). Briefly, the procedure includes pipetting 0.8 mL of enzyme reagents and 0.05 mL of specimen solution together into a clean dry test tube, before incubating for 5 min. Furthermore, 0.2 mL of starting reagent was added and mixed well, and the starting absorbance A0 at 340 nm was measured, and the absorbance was repeated every 1, 2, and 3 min. The average absorbance shift per time (A/min) was measured. The activity of SGPT (ALT) and SGOT (AST) was measured as U/L = A/min × 952.

### 2.6. Histopathological Studies

All the rats were euthanized at the end of the protocol, and the apex regions of the heart were excised and cleaned in an ice-cold saline solution. After washing, 10% buffered neutral formalin was utilized to immediately fix the cardiac tissue, after fixing tissues were mounted with paraffin, and histological evaluation was performed on cut 5 mm slices. Hematoxylin and eosin were used to stain paraffin slices. The stained slides were then studied under a light microscope to determine the degree of myocardial damage using the conventional procedure.

### 2.7. Statistical Analysis

The results are given as mean ± standard error mean (SEM). Graph-pad prism software, version 5.0, San Diego, California, USA, was used to perform the analysis. One-way ANOVA followed by Dunnett’s post-hoc test was used with *p* < 0.05 set as a significant level. Data were analyzed by one-way ANOVA followed by Dunnett’s post-hoc test (*p* < 0.05).

## 3. Results

### 3.1. Effect of TQ on Hemodynamic and ECG Parameters

Table 1 represents the modulation of arterial blood pressure by TQ. The ISO-treated rats had a substantial decrease (*p* < 0.001) in the SAP, MAP, and DAP compared to the normal. Two days of TQ treatment with ISO (TQ + ISO group) significantly reduced (*p* < 0.001) SAP, MAP, and DAP compared to the ISO group. Furthermore, the ISO caused a significant decrease (*p* < 0.001) in HR compared to the control. This effect of ISO was reversed by TQ, which is indicated by significantly restored HR function in TQ + ISO compared to ISO per se (*p* < 0.001). TQ alone did not cause any alteration in the SAP, DAP, MAP, and HR levels compared to normal.

### 3.2. Effects of TQ on Left Ventricular Function in ISO-Induced MI

**Figure 2:**
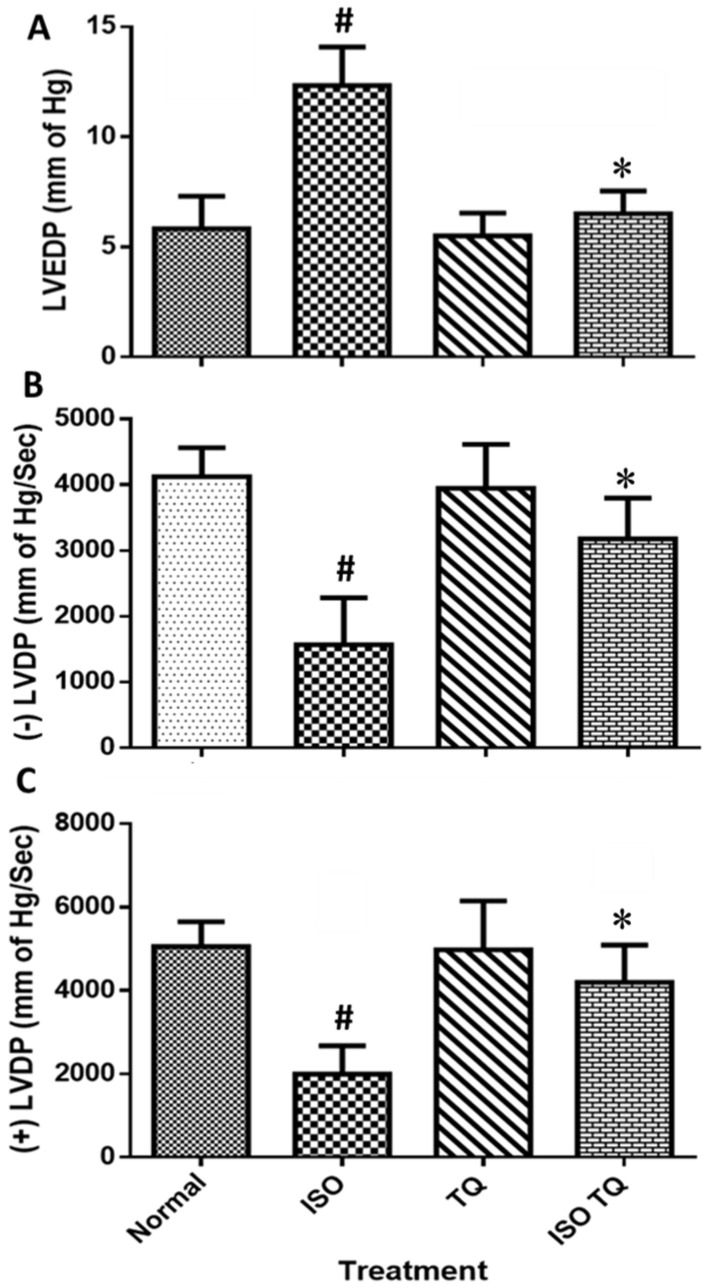
Effects of TQ on left ventricular function in ISO-induced myocardial infarction in rats. (**A**) LVEDP, (**B**) −LVdP/dtmax, and (**C**) +LVdP/dtmax. Data were analyzed by one-way ANOVA followed by Dunnett’s test. The data are presented as mean ± SEM (n = 6). ^#^ *p* < 0.001 vs. normal control; * *p* < 0.001 vs. ISO. ISO: isoproterenol; LVEDP: left ventricular end-diastolic pressure; -LVdP/dtmax: left ventricular maximum rate of negative pressure; +LVdP/dtmax: left ventricular maximum rate of positive pressure; TQ: thymoquinone.

Figure 2 illustrates the devastating effect of ISO on LVEDP and ±LVdP/dtmax, and the subsequent recovery to control values using TQ. The treatment of ISO caused ventricular dysfunction, as evidenced by a significant elevation in LVEDP (*p* < 0.001). Furthermore, compared to the control, ISO treatment lowered +LVdP/dtmax and -LVdP/dtmax (*p* < 0.001). TQ (TQ + ISO group) prevented an increase in LVEDP (*p* < 0.001) and improved LVdP/dt max and LVdP/dt min (*p* < 0.001) compared to ISO.

### 3.3. Effect of TQ on ECG in ISO-Induced MI

**Figure 3:**
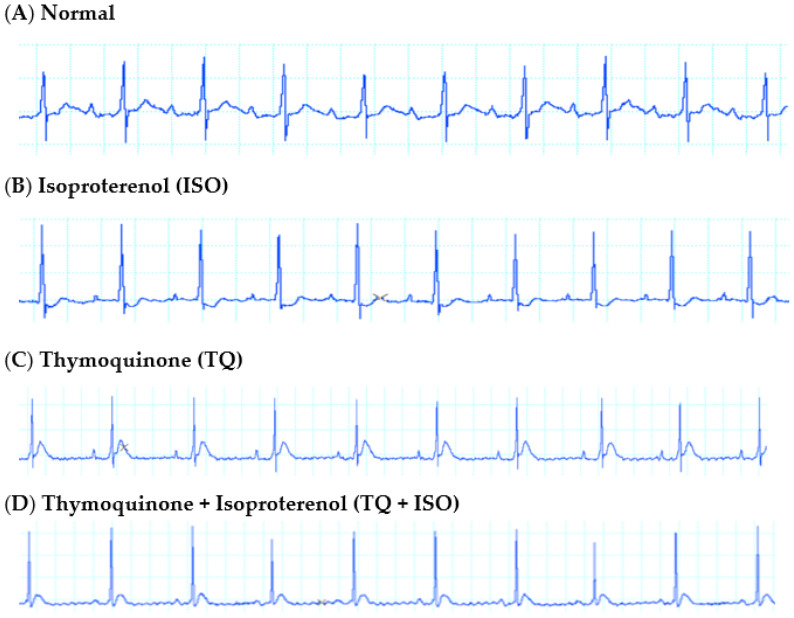
Representative lead II ECG tracings from rats receiving vehicle (**A**) normal; (**B**) ISO; (**C**) TQ; (**D**) TQ + ISO.

Figure 3 depicts weak ST-segment depression in the ECG waveforms following ISO treatment as compared to the normal control. Although TQ, per se, did not affect the ECG waveforms, its pretreatment (TQ + ISO) normalized the ECG waveforms as compared to the ISO group.

### 3.4. Effect of TQ on Oxidative Parameters in the Heart Tissues

**Figure 4:**
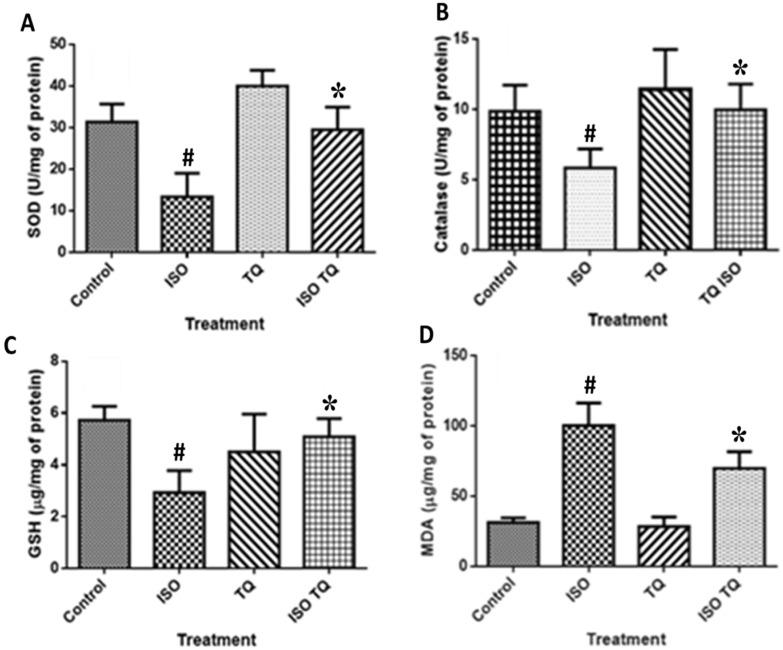
Effect of TQ on oxidative stress parameters (**A**) SOD, (**B**) CAT, (**C**) GSH, and (**D**) MDA in ISO-induced myocardial infarction in rats. Data were analyzed by one-way analysis of variance (ANOVA) followed by Dunnett’s post-hoc test. * *p* < 0.001 vs. normal control; ^#^ *p* < 0.001 vs. ISO. Data are expressed as mean ± SEM (n = 6). CAT: Catalase; GSH: glutathione; ISO: isoproterenol; MDA: malondialdehyde; SOD: superoxide dismutase; TQ: thymoquinone.

Figure 4 demonstrates that ISO injection produced adverse cardiovascular events; as a consequence, there is a remarkable decline (*p* < 0.001) in the SOD, CAT, and GSH levels when compared to the control group. ISO also induces lipid peroxidation which is demonstrated by increased activities of MDA as compared to the control group. TQ (TQ + ISO group) alleviated the negative effects of ISO by dramatically raising (*p* < 0.001) contents of both enzymatic (SOD and CAT) and non-enzymatic (GSH) antioxidants. Furthermore, TQ administration reduced the upsurge in lipid peroxidation caused by ISO, as demonstrated by lower (*p* < 0.001) MDA levels in the TQ + ISO group.

### 3.5. Effect of TQ on Myocardial Injury Enzyme Markers and Hepatic Enzymes

**Figure 5:**
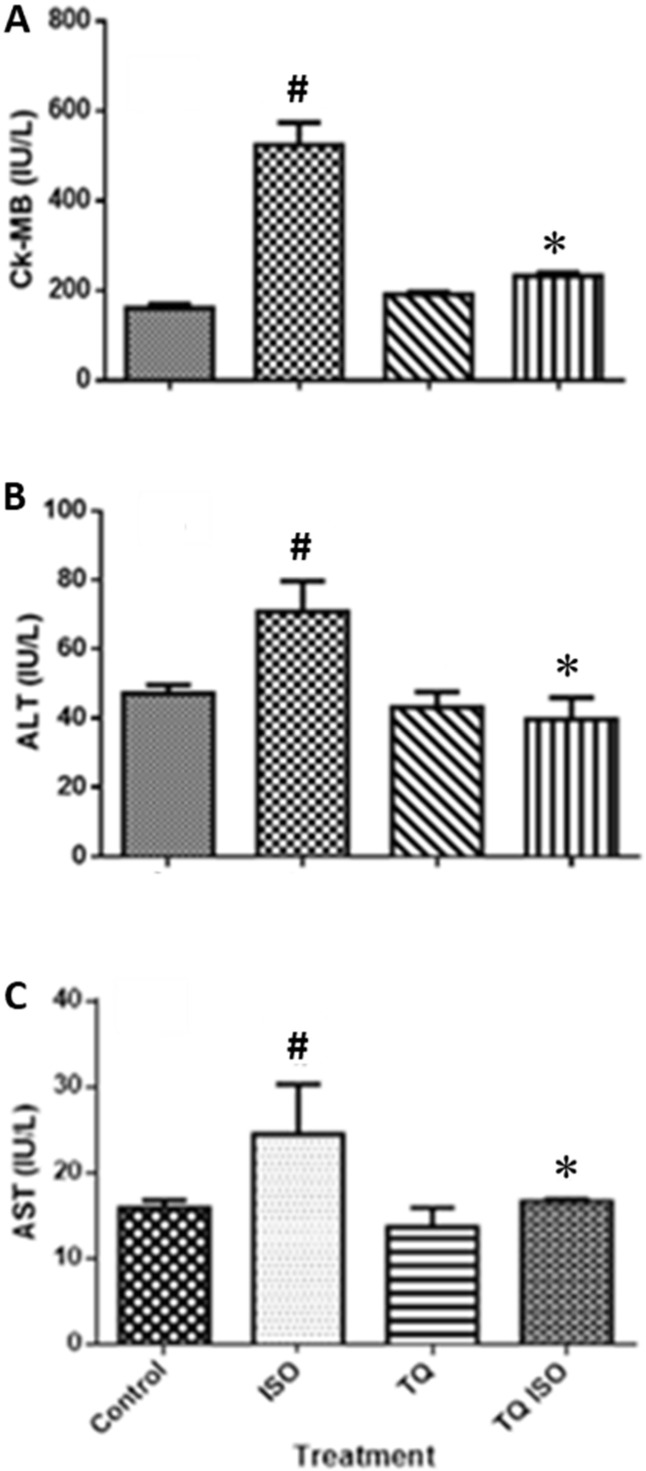
The effect of TQ on the serum level of cardiac marker enzymes (**A**) CK-MB, (**B**) ALT, and (**C**) AST in ISO-induced myocardial infarction in rats. Data were analyzed by one-way ANOVA followed by Dunnett’s test. * *p* < 0.001 vs. normal control; ^#^ *p* < 0.001 vs. ISO. The data are presented as mean ± SEM (n = 6). ALT: alanine aminotransferase; AST: aspartate aminotransferase; CK-MB: creatine kinase-MB; ISO: isoproterenol; TQ: thymoquinone.

Figure 5 depicts a substantial increase (*p* < 0.001) in myocardial injury enzymes such as CK-MB, ALT, and AST following ISO treatment compared to the normal. TQ treatments (TQ + ISO group) significantly attenuated (*p* < 0.001) the cardiac enzymes CK-MB, LDH, ALT, and AST compared to ISO treatment per se.

### 3.6. Effect of TQ on Proinflammatory Markers

**Figure 6:**
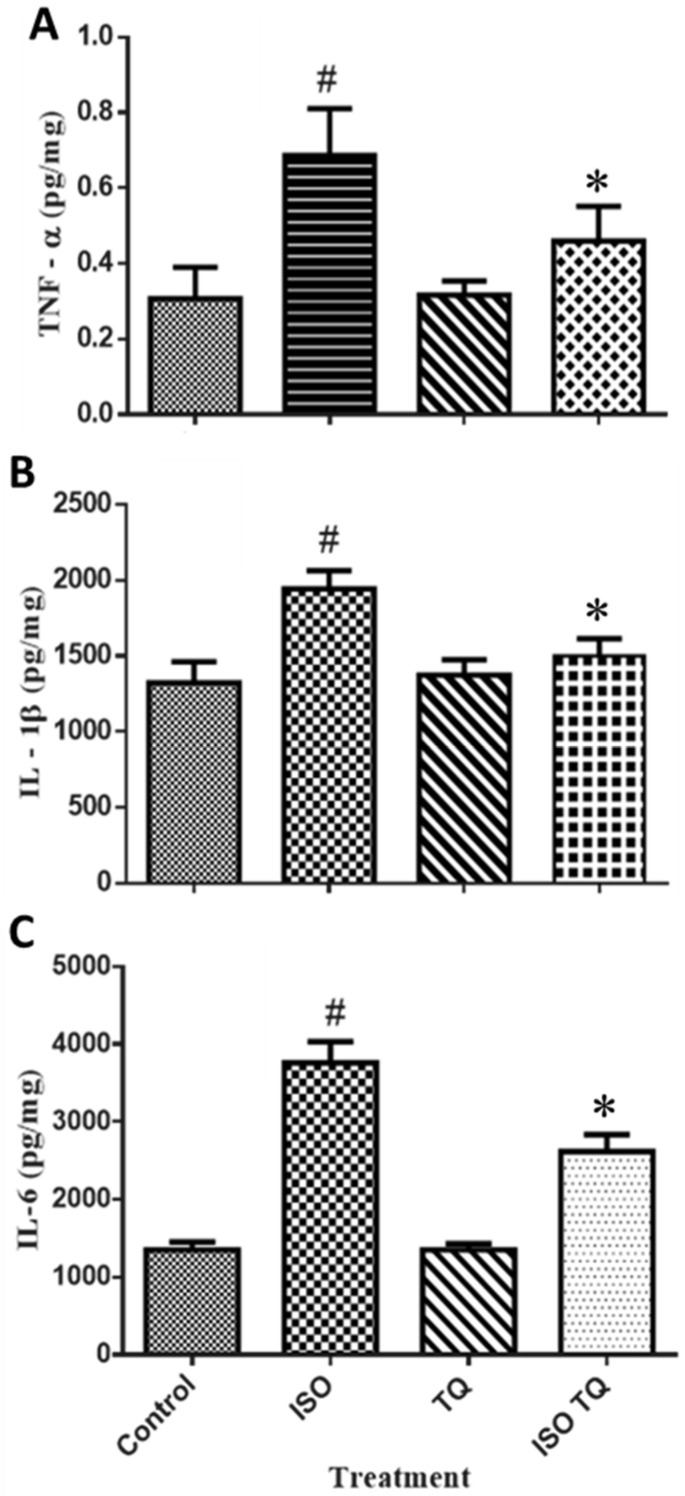
The effect of TQ on the proinflammatory biomarkers (**A**) TNF-α, (**B**) IL-1β, and (**C**) IL-6 in the myocardium. Data were analyzed by one-way ANOVA followed by Dunnett’s multiple comparisons test. * *p* < 0.001 vs. normal control; ^#^ *p* < 0.001 vs. ISO. The data are presented as mean ± SEM (n = 6). IL-1β: interleukin-1-beta; IL-6: interleukin-6; ISO: isoproterenol; TNF-α: tumor necrosis factor-alpha; TQ: thymoquinone.

Figure 6 shows the amounts of pro-inflammatory cytokines such as TNF-α, IL-1β, and IL-6. ISO-treated rats had a substantial (*p* < 0.001) rise in TNF-α, IL-1β, and IL-6 levels than the normal control. Two days of treatment with TQ remarkably decreased the levels of TNF-α, IL-1β, and IL-6 compared to the ISO group.

### 3.7. Effect of TQ on Histopathological Changes in the Heart Tissues

**Figure 7:**
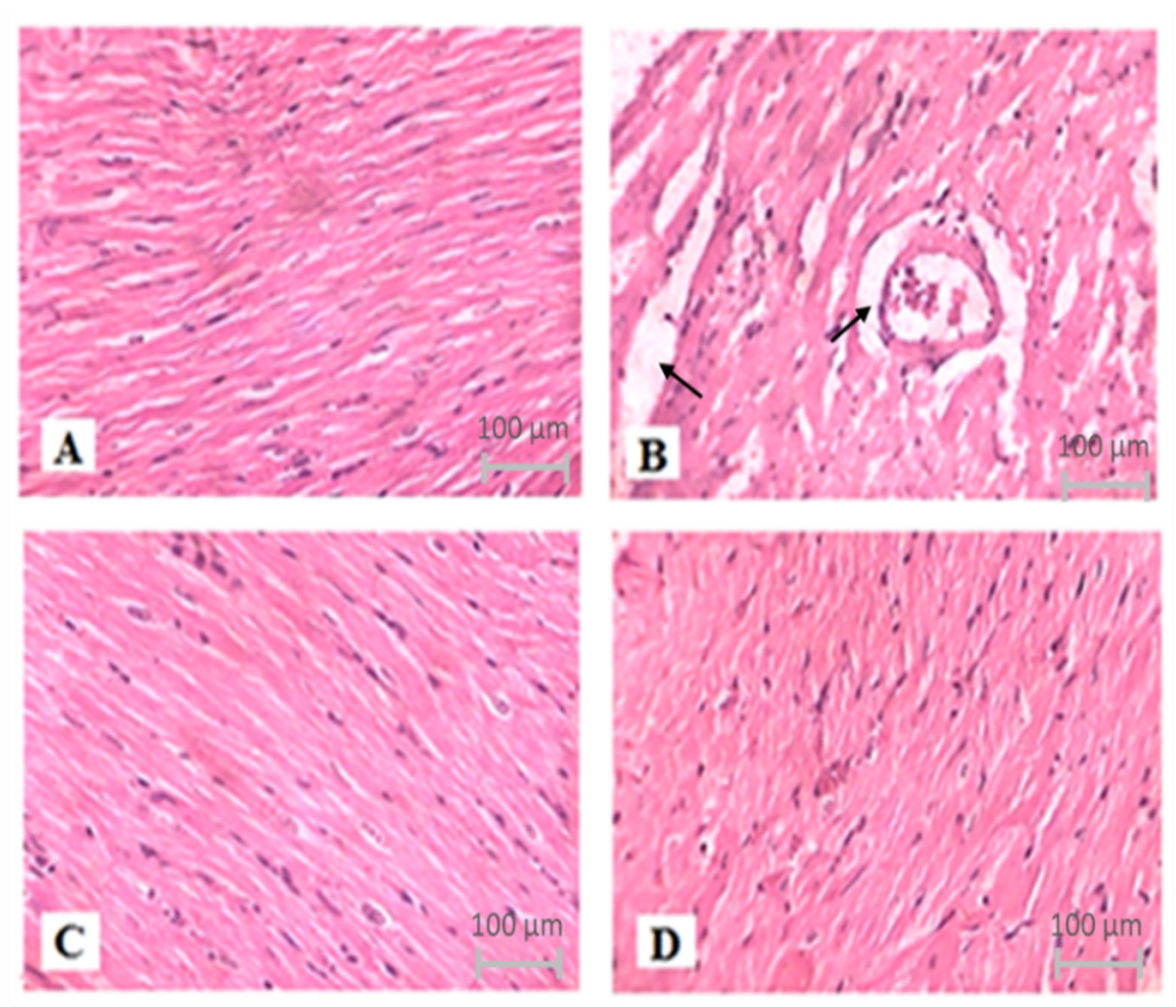
The effect of TQ on histopathological changes in ISO-induced myocardial infarction in rats. (**A**) Normal, (**B**) ISO, (**C**) TQ, and (**D**) TQ + ISO. Heart tissues were stained with hematoxylin and eosin and visualized under a light microscope at 400× magnification. ISO: isoproterenol; TQ: thymoquinone.

The degree of histological alterations in the myocardium of various groups is represented in Figure 7. Control groups showed normal architecture and no fraying or infarction (Figure 7A). The ISO group indicates localized confluent muscle fibers necrosis with infiltration of the inflammatory cell (Figure 7B). The impact of TQ alone was identical to the control (Figure 7C). TQ treatment of ISO-injected rats resulted in modest edema and a considerable decrease in infarction (Figure 7D).

## 4. Discussion

The current study demonstrates that acute TQ administration remarkably decreased the myocardial injury caused by ISO. ISO challenge showed deformation in ECG wave front, elevated LV end-diastolic pressure, and reduced LVdP/dtmax and LVdP/dtmin. The levels of the cardiac biomarker in serum such as CK-MB, ALT, and AST were increased. In the myocardium, a rise in MDA and decreased SOD, GSH, and CAT contents were observed. Furthermore, increased levels of TNF-α, IL-6, and IL-1β were observed in the myocardium. The SAP, DAP, and MAP in ISO-treated animals decreased as compared to control animals. The data of control and ISO-treated rats were in agreement with the earlier studies [30,31,32,33,34]. As anticipated, treatment with TQ used for normal animals did not affect any of the studied parameters in all the experiments. Interestingly, the effects of ISO were attenuated by TQ. Furthermore, TQ prevented alterations in the electrical functioning of the heart which postulates the protective ability of TQ against chemical (ISO)-induced MI in experimental animal models. The current investigation also found that TQ modulates oxidative stress against ISO-induced MI-like symptoms in rats. TQ substantially reduced TNF-α, IL-1β, and IL-6 levels. Our study also showed that TQ stabilizes the ST-segment. Previous studies postulated that ISO injection leads to morphological and functional changes in the myocardial tissue [35], which is well retained by treatment with TQ. ISO-induced MI is distinguished by myocardial dysfunction, lipid peroxidation, the reformed activity of cardiac biomarkers, and diminution of the heart’s natural oxidant/antioxidant equilibrium and antioxidant enzymes (notably SOD, CAT, and GSH).

As a consequence of ISO-induced MI, marked abnormalities in the cytokine levels, hemodynamic parameters, and structural parameters were observed. However, only a weak ST-segment depression in the ECG waveforms following ISO treatment was observed. Other investigations have found an increase in the ST segment in acute MI. When ISO was delivered, the magnitude of the T-wave reduced significantly. The T-wave represents ventricular repolarization; deviations may suggest electrolyte imbalance, left ventricular hypertrophy, or left bundle branch block [35,36]. Earlier data suggest that the electrical activity of the myocardium was significantly impaired by the necrosis induced by ISO [35]. In our study, we demonstrated that TQ treatment appears to improve myocardial electrophysiological function, primarily by attenuating the ISO-induced depression of the ST-segment. Together with electrophysiological alterations, ISO has an impact on both left ventricular and arterial blood pressure measures [31,32].

ISO also reduced arterial systolic and diastolic blood pressure, along with ventricular dP/dt min and dP/dt max. Since ISO generates peripheral vasodilation, the blood supply to the working myocardium is significantly reduced. This reduction in blood supply results in transient ischemia followed by myofibril necrosis. In the present model, this might be one of the developments that cause infarction and associated consequences such as oxidative stress. ISO-induced cardiac necrosis can lead to alterations in membrane permeability, resulting in a functional loss of myocardial membrane integrity [37,38,39].

In our study, we observed that TQ provides cardioprotection to the myocardium by positively affecting hemodynamic, biochemical, electrocardiographic, and histopathological parameters, indicating its protective effect on the myocardium. Similarly, current findings revealed that 2 days of treatment with TQ in ISO-treated animals leads to a remarkable decline in the blood pressure and heart rate. TQ has antioxidant and anti-inflammatory properties, as well as several biological benefits [38,40]. TQ impacts a wide range of enzymes involved in intracellular signaling. The current study found that ISO increased LVEDP in rats, which is a measure of pre-load, and implies improvement in left ventricular function. TQ reduces the rise in LVEDP caused by ISO treatment. By increasing inotropic (+LVdP/dtmax), a sign of myocardial relaxation (lusitropic), and (-LVdP/dtmax) contractility, TQ normalized left ventricular end-diastolic function. Our findings imply that TQ, through its multifunctional actions, protects against ISO-induced cardiac alterations.

The available literature demonstrates that the majority of the medicinal benefits of *Nigella sativa* are linked to the occurrence of TQ, a key pharmacological constituent of the essential oil [41]. Besides the hemodynamic, electrocardiographic, and ventricular functions, ALT and AST are known to represent pathological and internal changes in the myocardium, as well as serve as marker enzymes of myocardial injury [41]. During oxidative stress or myocardial damage, these enzymes are leaked from the cytosol into the extracellular fluid. In the current investigation, enzymatic activity was significantly reduced in ISO-challenged animals, indicating myocardial injury of the cardiac membrane.

TQ treatment for 2 days substantially reduced CK-MB, AST, and ALT levels during the ISO affront. TQ was tested for its influence on pro-inflammatory agents, such as TNF-α, IL-1β, and IL-6, where it showed a marked reduction in their levels. TQ was shown to be an effective anti-oxidant in the ISO-treated myocardium. Because of the decreased activity of antioxidant defense in cardiac tissues, the heart is extremely vulnerable to oxidative stress. In oxidative stress, these endogenous antioxidant compounds counteract ROS-mediated tissue damage [42]. It may be recalled that TQ has antioxidant and anti-inflammatory properties, as well as several biological benefits [39]. Following ISO administration, the reduction in SOD and CAT activity increases free radicals, superoxide, and hydrogen peroxide, and produces cellular damage. Following ISO administration, TQ therapy attenuated these alterations [43]. As a result, TQ is generally recognized as a powerful antioxidant that effectively protects against oxidative stress by raising GSH levels.

## 5. Conclusions

TQ, by restoring cardiac function, reducing oxidative stress, and suppressing the production of inflammatory cytokines, protects against myocardial injury triggered by ISO. The results are suggestive of the therapeutic and preventive potential of TQ in MI. However, further studies are needed to establish safety for human use and the first dose size for human studies, as well as to demonstrate the benefits in humans.

## Figures and Tables

**Table 1:** The effect of TQ on a hemodynamic parameter in ISO-induced myocardial infraction.

Groups	SAP (mm of Hg)	DAP (mm of Hg)	MAP (mm of Hg)	HR (Beats/Min)
Normal	119.6 ± 2.04	101.12 ± 1.65	110 ± 1.71	371.3 ± 3.33
ISO	92.71 ± 1.21 ^#^	75.3 ± 3.32 ^#^	84 ± 1.86 ^#^	245 ± 3.51 ^#^
TQ	111 ± 3.07	99 ± 4.30	105 ± 1.21	360 ± 2.85
ISO + TQ	112 ± 1.61 *	93 ± 3.02 *	102 ± 2.03 *	350 ± 2.92 *

Data are presented as mean ± SEM. ^#^ *p* < 0.001 vs. normal control group; * *p* < 0.001 vs. ISO. Data were analyzed using one-way ANOVA followed by Dunnet’s post-hoc test. SAP: systolic arterial pressure; DAP: diastolic arterial pressure; MAP: mean arterial pressure; HR: heart rate; ISO: isoproterenol; TQ: thymoquinone.

## Data Availability

Not applicable.
